# Improvement of the Immunity System Through Sports: Novel Regulatory Mechanisms for Hypertension

**DOI:** 10.31083/j.rcm2510385

**Published:** 2024-10-25

**Authors:** Jin Yang, Rui Sun, Zuowei Pei

**Affiliations:** ^1^Department of Central Laboratory, Central Hospital of Dalian University of Technology, 116033 Dalian, Liaoning, China; ^2^Emergency Department, The First Affiliated Hospital of Dalian Medical University, 116011 Dalian, Liaoning, China; ^3^Department of Cardiology, Central Hospital of Dalian University of Technology, 116033 Dalian, Liaoning, China; ^4^Faculty of Medicine, Dalian University of Technology, 116024 Dalian, Liaoning, China

**Keywords:** exercise training, immunity system, hypertension, inflammation, sports

## Abstract

Hypertension and its resulting target organ damage is a complex process associated with a range of physiological and molecular factors, including immune regulation. The profound effects of exercise on normal immune system function and the development and progression of hypertension are well known. This review aims to create new avenues for preventing and treating hypertension and its associated target organ damage. This narrative review emphasizes the role of exercise training in the prevention/treatment of hypertension development through immune response modulation and presents current perspectives on the available scientific evidence. Several studies have shown that exercise regulates hypertension by altering immune cells, which is partly attributable to the anti-inflammatory effects of exercise training. Regular exercise modifies immune modulation and could represent a new mechanism for regulating hypertension. Although the utilization of exercise training and the immune system in conjunction for treating and preventing hypertension is still in its early stages, current scientific literature indicates numerous potential physiological links between exercise training, the immune system, and hypertension.

## 1. Introduction

Hypertension is the leading risk factor for increased cardiovascular morbidity and mortality, accounting for up to 10.8 million annual deaths worldwide, or 19.2% of all attributable fatalities [1, 2]. Consequently, hypertension has become an important contributor to the global disease burden in developed and developing countries. European Society of Hypertension (ESH)/European Society of Cardiology (ESC) proposes a cut-off point of systolic blood pressure (SBP) ≥140 mmHg and/or diastolic blood pressure (DBP) ≥90 mmHg to identify hypertension [3]. Currently, effective treatment of hypertension requires a variety of modalities: proper diet, exercise, medication control, and so on [4]. However, monotherapy is difficult and usually requires combination therapy [5].

The human immune system is a defense network that covers the entire body, and daily factors determine its many reactions. In addition to uncovering the connection between the transient activation of the sympathetic nervous system (SNS), the renin-angiotensin-aldosterone system (RAAS), and the dysregulation of vasoactive substances with the progression of hypertension, Navaneethabalakrishnan S and colleagues have demonstrated the escalating significance of immune cells in the pathogenesis of hypertension [6].

The importance of regular physical exercise for people with essential hypertension has emerged as a major modifiable factor contributing to optimal blood pressure control. Aerobic exercise benefits the cardiovascular system by promoting traditional cardiovascular risk factor regulation and favorably regulating SNS activity, vasoactive substances, and RAAS. Acanfora *et al*. [7] found that exercise training enhances cardiac function indices and lung function early after acute cardiac decompensation episodes in middle-aged and older patients with heart failure. Regular exercise significantly benefits the immune system and patients with chronic diseases, including hypertension [8, 9]. Comprehensive research has revealed a strong link between exercise, immune cells, and hypertension. Exercise training can change white blood cells, red blood cells, and cytokines to control the immunological response, which can further influence the development and progression of hypertension [10].

Simultaneously, exercise produces an immunological response, such as creating cytokines, which contributes considerably to controlling local and systemic inflammation and improves the immune response. As highlighted, inflammation is a critical component of the human immune response and is important in immunological control. There is a consensus on the involvement of inflammation in the pathogenesis of hypertension, which is frequently associated with suboptimal blood pressure control in hypertensive patients [11]. Nevertheless, exercise has extra complexity for inflammatory effect, and a growing body of research now shows that exercise may attenuate the action of inflammatory mediators and improve the anti-inflammatory context in the body [12, 13].

The intensity, duration, and form of exercise considerably impact immune system function [14]. Moderate-intensity continuous training (MICT) is thought to have many benefits, while high-intensity interval training (HIIT) can induce inflammation and reduce cell-mediated immune system function [15, 16, 17, 18]. The routine practice of MICT directs the immune response to an anti-inflammatory state, which is thought to be the primary mechanism through which exercise improves health [8]. Meanwhile, moderate aerobic exercise such as yoga, walking, jogging, and swimming is thought to play an important role in reducing inflammation [19]. A growing body of evidence suggests that dynamic resistance training may affect blood pressure similarly to aerobic training [20]. This review utilizes various *in vitro* and *in vivo* models to describe potential mechanisms through which exercise training further controls the pathology of hypertension progression by exerting its immune-modulatory effects.

## 2. Immunity System Activation in Hypertension

The immune system covers all organs and is responsible for identifying and eliminating external objects, foreign pathogenic microbes, and other elements that can disturb the body’s internal environment. Innate and adaptive immunity are two branches of the immune system that play an indisputable role in the initiation and progression of chronic inflammatory diseases, such as hypertension [21, 22].

Most immune cells are involved in developing, progressing, and maintaining hypertension in addition to the traditional non-immune factors that elevate blood pressure, such as high salt intake, angiotensin Ⅱ (Ang Ⅱ), aldosterone, catecholamines, and impaired renal water–sodium exchange [23]. Numerous immune cell subsets have been shown to invade blood pressure-regulating organs such as blood arteries, kidneys, the heart, and the brain during hypertension, and targeted depletion of particular immune cell subsets has been shown to prevent hypertension in animal tests [24, 25]. Inflammation is also associated with poor blood pressure control in hypertensive patients [11]. Immunology linked to the onset of hypertension and hypertensive end-organ damage will be discussed in detail in the following sections, along with suggested mechanisms for how they influence hypertension.

Research has found that in the presence of genetic redisposition, environmental factors such as salttrigger SNS activation and parasympathetic nervous system (PNS) suppression [26], either alone or in conjunction with other hypertensive stimuli such as Ang Ⅱ, aldosterone, and endothelin-1, resulting in small increases in blood pressure. As the illness worsens, hypertension and/or pro-hypertensive stimulation cause tissue damage, which, along with oxidative stress brought on by vasoactive peptides, such as Ang Ⅱ or endothelin-1, encourages the formation of damage-associated molecular patterns (DAMPs) and neoantigens and further triggers subsequent inflammatory responses. While neoantigens enhance the immune response of dendritic cells (DCs), leading to T cell proliferation and the release of inflammatory cytokines, DAMPs activate innate immune responses by interacting with Toll-like receptors (TLRs).

**Fig. 1.**
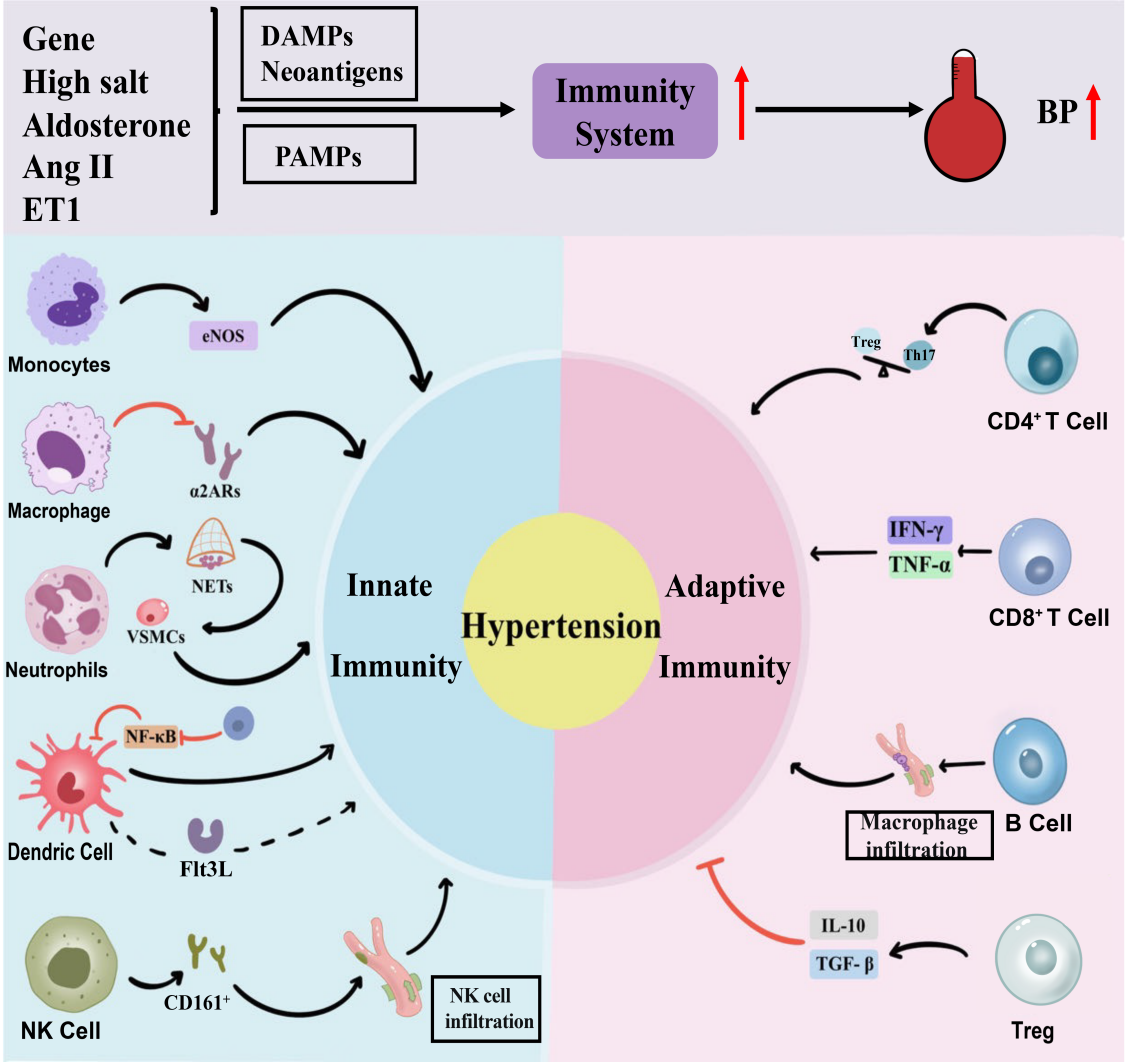
**The role of immune cells in hypertensive inflammation**. ET-1, (endothelin, ET)-1; DAMPs, damage-associated molecular patterns; PAMPs, pathogen-associated molecular patterns; BP, blood pressure; eNOS, endothelial nitric oxide synthase; α2Ars, α2-adrenergic receptors; NETs, neutrophil extracellular traps; VSMCs, vascular smooth muscle cells; Flt3L, fms-like tyrosine kinase 3 ligands; Treg, regulatory T cells; NK cell, natural killer cell; NF-κB, nuclear factor kappa-B; Th17, T helper cell 17; Ang II, angiotensin II; IFN-γ, interferon-γ; TNF-α, tumor necrosis factor-α; IL-10, interleukin-10; TGF-β, transforming growth factor-β.

Additionally, pathogens (bacteria, viruses, fungi, etc.) can sense the pathogen-associated molecular patterns (PAMPs) by attaching to pattern recognition receptors such as TLRs, triggering inflammatory or antimicrobial responses to increase the activation of innate immunity. DAMPs may also initiate adaptive immunity through major histocompatibility complex Ⅱ (MHC Ⅱ) located on antigenic presenting cells (APCs), drive T and B lymphocyte activation leading to inflammation, induce pro-inflammatory cytokines, and produce autoantibodies leading to vascular and kidney damage, a feedforward process that leads to progressive blood pressure increases (Fig. 1).

### 2.1 Innate Immunity 

The first line of defense for the host against danger signals is provided by innate immune responses, which are present at birth and function in a wide, quick, generally stable, and nonspecific manner [27]. Innate immune responses are frequently described as the first stage of inflammation. Monocytes, macrophages, neutrophils, and dendritic cells that develop from common myeloid progenitor cells (CMPs) in the bone marrow, as well as natural killer cells that develop from common lymphoid progenitor cells (CLPs), are the main contributors. Conversely, α2-adrenergic receptors (α2ARs) inhibit the secretion of norepinephrine. In deoxycorticosterone acetate (DOCA)-induced hypertension animal models, it has been demonstrated that decreased macrophages can preserve the function of α2ARs and lower blood pressure [28].

Loperena *et al*. [29] demonstrated that Ang Ⅱ-induced increases in hypertension and vascular dysfunction can be avoided by inhibiting monocyte production and transformation to other cell types *in vivo*, while attenuating endothelial nitro-oxidative stress and endothelial nitric oxide synthase (eNOS) uncoupling. A significant role in innate immunity is played by neutrophils, which can infiltrate target organs, release pro-inflammatory chemicals and vasoactive neurotransmitters, and initiate hypertension-related inflammatory states [30, 31]. It is believed that neutrophils may contribute to the onset and progression of hypertension through neutrophil extracellular traps (NETs) [32]. Neutrophils can create NETs in response to persistent inflammation, which causes particular types of cell death. NETs, which promote vascular remodeling by inducing phenotypic changes in vascular smooth muscle cells (VSMCs) , have positively correlated with blood pressure, making them a prospective target for anti-hypertensive therapy [33, 34]. In addition, a recent study has found that neutrophil-to-lymphocyte ratios and neutrophil counts are associated with an increased risk of developing hypertension [31].

APCs in the body can be efficiently taken up, processed, and presented by DCs, the most specialized and functioning APCs in the body, thus performing vital roles in developing, controlling, and maintaining the immune response. A growth factor called Fms-like tyrosine kinase 3 ligands (Flt3L) promotes the formation of traditional DCs [35]. Notably, Lu *et al*. [36] found considerably fewer classical DCs in Flt3L-deficient mice. The hypertensive response and T-cell activation in the kidneys of these mice both decreased at the same time. Another study found that the ubiquitin-editing protein A20 in CD11c+ myeloid cells may further reduce T cell activation by inhibiting the activation of the nuclear factor kappa-B (NF-κB) signaling pathway and inhibiting dendritic cell maturation, thereby inhibiting the inflammatory response that causes hypertension when RAS is activated. A further previous study has indirectly shown that dendritic cells have a potential relationship with the occurrence and development of hypertension [37].

Natural killer (NK) cells are essential immune cells in the body, which serve as the first line of defense against malignancies and viral infections and significantly impact the control of the immune system. NK cells can remove atherosclerotic plaques in blood vessel walls and impurities in blood, purifying the blood, expanding blood vessels, and accelerating hemorheology, thus reducing blood pressure and improving cardiovascular and cerebrovascular system functions. According to relevant study, immune complexes accumulate in blood vessels to form thrombosis, increasing blood pressure [38]. NK cells can clear immune complexes in blood vessels and stabilize blood pressure within normal values, thus effectively preventing hypertension complications.

### 2.2 Adaptive Immunity

#### 2.2.1 Immune Responses Determined *In Vitro* and *Ex Vivo*

The role of an engaged adaptive immune system in driving up hypertension while escalating damage to target organs has been amply documented in recent years. The adaptive immune response, known as specific immunity, is a delayed secondary antigen-specific response, which is primarily controlled by activated T and B cells and engages in many different immune responses in the body, including multicellular participation, antigen memory retention, and specific recognition [39]. T lymphocytes are primarily divided into CD4^+^ and CD8^+^ subtypes. Data from *in vivo* studies imply that CD4^+^ T cells may have an important role in influencing the development of hypertension, even though both types of T lymphocytes have been demonstrated to be major contributors to the pathogenesis of hypertension [40, 41].

However, according to a study by Lu *et al*. [37], prehypertensive cytokines generated from CD8^+^ T cells may be a major contributor to hypertension. CD8^+^ T cells mature and secrete interferon-γ (IFN-γ) and tumor necrosis factor-α (TNF-α), leading to increased inflammatory response, another way of inducing hypertension [37]. T cells respond to an endogenous or foreign antigen by activating and differentiating into T effector cells. Therefore, through regulating inflammatory mediators such as cytokines and chemokines, activated T lymphocytes migrate to the site of inflammation and perform cellular immune responses [42]. Numerous experimental studies have shown that several T cell subsets can promote vascular remodeling and hypertension [37, 40]. Ang Ⅱ induces hypertension to some extent through adaptive immune and effector T lymphocyte regulatory mechanisms, and Treg can suppress effector T lymphocytes through interleukin (IL)-10 or Transforming growth factor-β (TGF-β). Therefore, Treg can somewhat suppress Ang Ⅱ-mediated vascular damage through anti-inflammatory effects, confirming the involvement of immune mechanisms in Ang Ⅱ-induced blood pressure elevation, vascular peroxidative stress, inflammation, and endothelial dysfunction [41]. Treg adoptive transfer attenuated Ang Ⅱ-induced systemic inflammation, hypertensive responses, and cerebrovascular damage in an Ang Ⅱ-induced hypertension animal model [43]. The balance of T cell subsets may influence the inflammatory response. Vascular remodeling and cardiac hypertrophy are primarily brought on by T helper cell 17 (Th17) cell and Treg cell imbalance, according to the study by Imiela *et al*. [44], which described a link between CD4^+^ T cell imbalance and other factors, including hypertension and target organ damage. According to recent studies, Th17 cells, a crucial component of effector T cells, may also increase blood pressure [45, 46]. In addition, Kim *et al*. [47] discovered that in juvenile-onset hypertensive rats (SHRs), the transfer of Th17 cells enhanced the proportion of CD4^+^IL-17A^+^ (Th17) cells and quickened the development of hypertension. In conclusion, Th17 cells trigger immunological responses, which results in endothelial dysfunction and causes hypertension to develop and worsen while hastening damage to many target organs. The prevention and management of hypertension are greatly enhanced by inhibiting Th17 cells, which also improves ventricular hypertrophy and remodeling and reduces further immune and inflammatory response activation.

There is a consensus that T cells play a key role in hypertension; however, a role for B cells has also recently been proposed. According to Dingwell *et al*. [48], mice with B cell depletion have lower blood pressure. In addition, infusion of Ang II into B cells activating factor knockout mice revealed that aortic macrophage infiltration was reduced, collagen deposition and sclerosis were prevented, and elevated blood pressure was attenuated [49]. Altogether, these results suggest that B cells may contribute to elevated blood pressure and produce a pro-inflammatory environment that leads to vascular injury.

#### 2.2.2 Immune Responses Determined *In Vivo*

To overcome limitations associated with *in vitro* experiments that assess the involvement of immune cells in the course of hypertension, several studies have investigated the influence of immune cells on the onset and development of hypertension. Sereti *et al*. [23] undertook a comprehensive assessment of 61 consecutive hypertension patients and 55 healthy individuals of similar age and gender distribution and generated a complete immunological profile, including quantifying immunoglobulin (IgG, IgM, IgA) and lymphocyte subsets. The levels of immunoglobulin IgG, IgA, IgM, and complement factor C3 in hypertension patients were considerably greater than in the control group. These data could support the idea of altered cellular and humoral immune responses in the pathophysiology of hypertension [23]. Meanwhile, an observational cross-sectional case–control study compared 105 patients with noncomplex and otherwise healthy hypertension: a total of 53 with well-controlled blood pressure and 52 with uncontrolled blood pressure. Arterial hypertension stimulates the immune response regardless of the state of blood pressure regulation. Chronic hypertension affected peripheral monocyte Toll-like receptor 4 (TLR4) expression and IL-17A serum level, while there was a correlation between the IL-17A concentration and the duration of hypertension [50]. This study provides a new direction for the prevention and treatment of hypertension in the future.

### 2.3 Immunosenescence

Aging is a normal physiological process in which the function of various organs gradually changes with age; when it affects the human immune system, it is known as immunosenescence. The human immune system protects people from environmental stress and other biological threats. However, in the aging state, the immune system typically exhibits a relatively constant low level of activation; when stimulated by the outside world, its dynamic response weakens, and the amplitude decreases, and this combination of chronic inflammation and reduced effective defense ability is commonly referred to as immunosenescence [51]. Chronic systemic inflammation is a feature of immunosenescence. Low inflammation is a pathogenesis of many age-related diseases, such as atherosclerosis, hypertension, Alzheimer’s disease, osteoporosis, etc.

Hypertension has a high incidence in the older adult population, which is at least partly related to a series of adverse remodeling of the cardiovascular system caused by immune aging. The pathogenesis of some cases is related to atherosclerosis. For example, secondary hypertension may result when blood vessel plaques in the body cause blood flow obstructions in the kidney arteries. In experimental hypertension, Ang Ⅱ induces chronic inflammation of blood vessels by activating T cells to produce TNF-α, leading to increased blood pressure. Blocking TNF-α downstream signaling prevents hypertension and Ang Ⅱ-induced increases in vascular reactive oxygen species (ROS) production [52]. In summary, therapies targeting the regulation of inflammatory aging-related factors can prevent or alleviate hypertension to a certain extent. The frequency of CD8^+^CD28^–^ or CD8^+^CD57^+^ T cells in the peripheral blood of patients with essential hypertension increases, which are senescent T cell phenotypes. In addition, in the peripheral blood of hypertensive patients, the number of CD8^+^ T cells that produce IFN-γ and TNF-α increased [52, 53]. This clinical phenomenon can be partly explained by the process of immunosenescence, whereby continuous antigenic stimulation leads to the loss of CD28 and the increase in CD57 on the surface of T cells, resulting in the accumulation of senescent T cells. These cells promote the proliferation and thickening of vascular endothelial cells and the release of vasoactive substances by releasing pro-inflammatory cytokines and cytotoxic mediators, leading to hypertension [53, 54]. However, it is unclear whether aging T cells cause essential hypertension or simply a concomitant of the disease. Simultaneously, the specific reasons for the accumulation of senescent T cells remain to be studied.

## 3. Exercise Training Regulates the Immune System in Hypertension Patients

Lifestyle changes are seen as an important first line of defense in treating people with high blood pressure, and exercise is considered a key component of treatment. According to our above content can be found that exercise can alter cell-mediated immune system activity and influence the onset and course of hypertension by producing anti-inflammatory cytokines at the injury site. Interestingly, exercise intervention for hypertension resulted in an average blood pressure reduction of 16/11 mmHg, depending on the intensity and frequency of exercise [8, 9, 14]. The relationship between the frequency and duration of exercise and its effect on blood pressure is complex.

### 3.1 The Effects of Exercise on the Innate Immune Response in Hypertension Patients

#### 3.1.1 Exercise Training and Monocytes/Macrophage

It has been demonstrated that macrophages play a significant role in the development of hypertension by regulating water and salt metabolism in the kidneys [55]. Exercise training may diminish macrophage infiltration into other chronic inflammatory target organs, such as the kidneys [56]. Meanwhile, regular exercise minimizes inflammation caused by renin-angiotensin system (RAS), reduces the number of inflammatory monocytes/macrophages in the blood at rest, and guards against SNS activation and hypertension. Recent research by Cooper and Radom-Aizik [57] has demonstrated that MICTs influence the monocyte gene pathways, which limit pro-inflammatory monocytes and reduce vascular damage. Additionally, an examination of 31 prehypertensive individuals with high blood pressure found a moderate impact of regular exercise on the phenotype of immune cells [58]. According to a cross-sectional study, women who engaged in acute moderate-intensity exercise training decreased monocyte chemokine receptor 2 (CCR2) expression, which increased the polarization of anti-inflammatory macrophages (M2) and reduced the polarization of pro-inflammatory macrophages (M1) [58]. Overall, MICT influences blood pressure by altering the central sympathetic nervous system, water and sodium metabolism, and the local inflammatory response of blood vessels.

#### 3.1.2 Exercise Training and Neutrophils

Regular moderate-intensity exercise promotes human health by influencing neutrophils and lowering inflammation severity. Because neutrophils are strongly related to the etiology of hypertension, we predicted that regulating neutrophils could also somewhat delay the development of hypertension. Regular physical activity has been shown to reduce vascular injury and remodeling caused by neutrophil-mediated inflammatory responses, including hypertension prevention and treatment, by increasing DNase activity, thus limiting the ability of NETs to participate in pro-inflammatory signaling [59]. Furthermore, HIIT treatment in 12 sedentary males promoted the late activation of neutrophils through the production of reactive oxygen species and the activity of superoxide dismutase, and changes in reactive oxygen species are also associated with human susceptibility to infection [60]. While the physiological significance of exercise-induced changes in neutrophil function remains open to study, the overall trend is consistent with epidemiological evidence that vigorous exercise increases susceptibility to infection, while moderate exercise may enhance immunity. On the other hand, MICT can inhibit the function of certain neutrophils and may reduce the likelihood of neutrophil-mediated inflammatory tissue damage.

#### 3.1.3 Exercise Training and Dendric Cells

Exercise training-induced DC mobilization may play a role in treating and preventing hypertension. DCs recognize and present isoketal to T cells, causing their activation and mediating the inflammatory response. Therefore, recognizing and presenting hypertensive irritants as antigens by innate immune cells to acquired immune cells are key steps in causing hypertension. The B7 ligand CD86 was significantly increased, which induced DCs to form isolevuglitide adducts (IsoLGs) and stimulated T cells to produce pro-inflammatory factors, causing hypertension [35, 61]. One study has found that aerobic exercise slows down DC maturation and activation and helps to reduce the release of pro-inflammatory factors and the occurrence of inflammatory responses [61]. At the same time, Flt3L, a growth factor that stimulates DC development, was identified through a cross-over study to significantly reduce Flt3L expression with single and especially repetitive grip exercises [62]. It also indirectly suggests that exercise training reduces the ability of dendritic cell overactivation to cause hypertension.

#### 3.1.4 Exercise Training and Natural Killer Cells

Exercise training greatly affects NK cells, and regular MICT is associated with increased cytotoxic activity of NK cells. Moreover, exercise can increase the number and activity of NK cells, which may be due to catecholamine-mediated and cell aggregation effects [63, 64]. However, HIIT can lead to neutrophil respiratory bursts and reduced NK cell activity [65, 66]. In addition, athletes who undergo high-intensity training over a long period may also have adaptive immune damage, and the NK cell function is significantly reduced. In general, NK cells depend on exercise time and intensity. Therefore, exercise should be rationally arranged and designed according to the influence of NK cells to control hypertension.

### 3.2 The Effects of Exercise on Adaptive Immunity in Hypertension

#### 3.2.1 Exercise Training and T Cells

Several studies have found that these changes in T cell numbers by exercise may be proportional to the intensity and duration [14, 67]. The MICT protocol appears to promote immune regulation, such as reducing T cell proliferation, restoring the balance between T cell subsets, and contributing to the negatively regulated inflammatory profile of hypertensive individuals [67]. T cell function appears to be sensitive to increased training load. In animal models, appropriate and regular running training corrected middle cerebral artery occlusion (MCAO)-induced Th17/Treg imbalance and reduced pro-inflammatory cytokine concentrations [68]. Fernandes *et al*. [69] found in a mouse model of allergic asthma that physical exercise significantly increased anti-inflammatory cytokine production and increased the recruitment of M2 in the lung, as well as the influx and activation of Tregs and CD4 and CD8 lymphocytes. These findings suggest that physical exercise regulates allergic inflammation by increasing Treg and M2 recruitment, which leads to an increase in anti-inflammatory cytokines and a decline in pro-inflammatory cells and mediators. In a murine model of Ang Ⅱ-induced hypertension, MICT resulted in a significant decrease in chemokine (C-C motif) receptor 5 (CCR5) and CD25 expression on CD8+ T cells infiltrating perivascular adipose tissue [51]. In conclusion, the above studies suggest that MICT has a moderate regulatory influence on the function and expression of individual immune cells that ultimately act on the pathophysiological events leading to hypertension.

#### 3.2.2 Exercise Training and B Cells

After immune activation, B cells proliferate and differentiate, maturing into memory cells and plasma cells. It has been suggested that the number of B cells increases mildly during and immediately after exercise and is proportional to the duration and intensity. Exercise plays an important anti-inflammatory role by upregulating the Fc gamma receptor IIB expression in B cells [70]. In addition, a sustained increase in circulating B cell numbers was detected during or after high-dose resistance exercise, while elevated circulating B cell counts were detected even during low-dose resistance exercise. However, as there are no clear conclusions regarding the mechanisms involved in the B cell regulation of hypertension, further studies are needed to elucidate the effects of exercise training on B cell immune function and its role in the pathogenesis of hypertension.

### 3.3 Various Types of Exercise Training Affect Hypertension Through the Immune System

In addition to the intensity and duration of the exercise, several types of exercise have been shown to impact the immune system and hypertension. Despite a lack of relevant data, many linkages may be established between available studies and known effects of physical activity, implying that exercise modification of the immune system function may be a significant modulator of hypertension. It was discovered that the 5^′^ adenosine monophosphate-activated protein kinase (AMPK) is used in the immune system to limit inflammatory activation in macrophages, DCs, and T cells [71]. Moreover, using an animal model, Nazari *et al*. [72] discovered that swimming corrected the decrease in AMPK production by experimental autoimmune encephalomyelitis. Furthermore, exercise boosted adenosine 5‘-monophosphate (AMP)-activated protein kinase (AMPK)/sirtuin 1 (SIRT1)/ peroxisome proliferator-activated receptor gamma coactivator-1 (PGC-1)/recombinant forkhead box protein O3 (FOXO3) pathway protein expression when combined with the DIKTNKPVIF (DF) peptide to reduce hypertension in SHR [73]. The Th1/Th2 ratio, particularly Th1 cells, is connected to the inflammatory response. According to research, the Th1/Th2 immunological imbalance persists for at least one week after intense activity, such as marathon running, considerably increasing the risk of inflammatory reactions and other disorders mediated by inflammation, such as high blood pressure [74]. Because older individuals are more likely to develop hypertension, age-related changes in the immune system increase the risk of cardiovascular disease. Regular physical activity may assist in preventing the onset and progression of several chronic diseases, including cardiovascular disease, hypertension, and cognitive impairment in older adults. A research of 29 older women with inactive lifestyles (mean age: 67.03 ± 3.74 years) discovered that subjects were given 60 minutes of functional activity, such as running, stationary bicycles, and squats, for 6 weeks. After the exercise program, CD8+ T cell numbers fell, and the distribution of CD8+ T cell subsets changed significantly [75]. The inflammatory response is the key regulatory mechanism through which excessive immune system activity exacerbates hypertension. A 4-month study on the effects of yoga on industrial workers found a decline in the pro-inflammatory factor IL-1β and a boost in the anti-inflammatory factor IL-10, indicating that yoga has an anti-inflammatory impact on those who encounter contaminants and inflammatory conditions [19]. A recent meta-analysis of the impact of yoga on immune function discovered that yoga lowered inflammatory cytokine (IL-1β) levels in both healthy and clinical populations. Furthermore, no research found a rise in pro-inflammatory markers or a reduction in anti-inflammatory markers, implying that yoga may not harm immune function [76]. In conclusion, most exercise regimens have favorable impacts, and regular exercise benefits a variety of systemic disorders and organ functioning. However, no consensus remains on these benefits, and the relevant intervention mechanisms must be studied more in the future. Moreover, we must examine more subjects and establish appropriate control groups for further validation.

## 4. Novelty

Although the immune system and its role in the pathogenesis of hypertension have been the focus of previous research, there is also considerable interest in how exercise training may modulate the immune system and affect the development and progression of hypertension. In this article, we describe the effects of different intensities and forms of exercise on the immune response and further discuss the mechanisms through which to intervene and treat hypertension. At the same time, we briefly discuss their potential health and clinical implications, providing novel perspectives for the treatment and prevention of hypertension.

## 5. Limitation

However, the role of certain factors associated with the induction and progression of hypertension, such as the complement system, oxidative stress, cytokines, chemokines, and MHC in the immunological contribution to disease progression and how they are modulated by exercise training, has not been extensively described in this review. The impact of exercise training in modulating the immune system and its role in hypertension can be further elucidated in the future due to the complex interactions between these systems.

## 6. Conclusions 

**Fig. 2.**
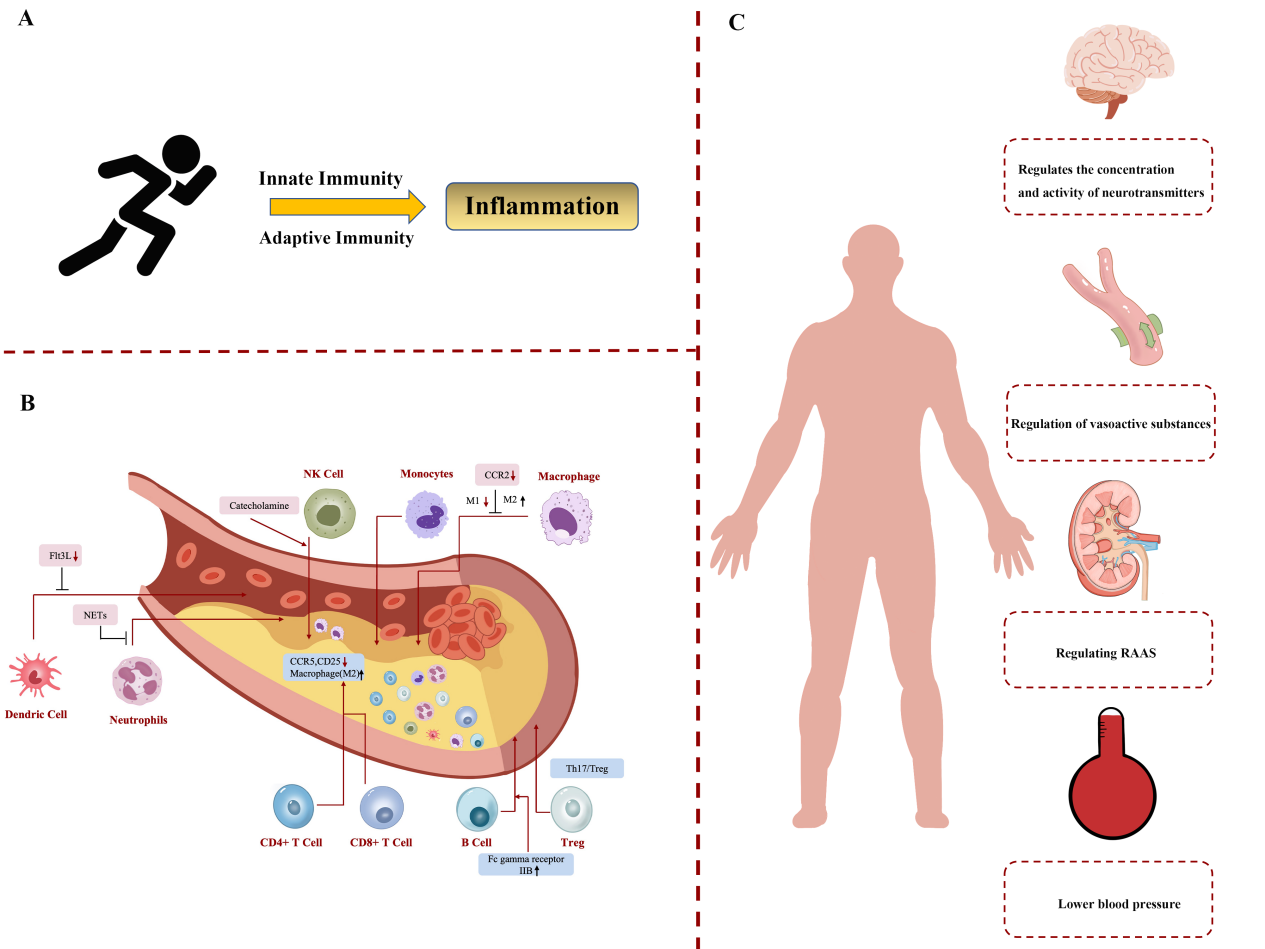
**Exercise training reduces hypertension and targets organ damage by regulating the immune system**. (A) The relationship between exercise training and immune system. (B) The mechanism of action of exercise training on different immune cells. (C) Exercise training regulates the mechanism of hypertension through the immune system. Flt3L, fms-like tyrosine kinase 3 ligands; NETs, neutrophil extracellular traps; NK cell, natural killer cell; CCR2, chemokine receptor 2; M1, macrophages; M2, macrophages; Treg, regulatory T cells; RAAS, renin-angiotensin-aldosterone system; Th17, T helper cell 17.

Current research has proved that exercise is the cornerstone of managing, preventing, and treating hypertension and its associated comorbidities. It is widely recognized that the effects of proper exercise on normal body function are profound. During exercise, the development and maintenance of hypertension are regulated by influencing immune cells and inflammatory responses (Fig. 2). In essence, this points to a new direction for the prevention and treatment of hypertension and target organ damage, with the possible goal of using exercise training as one of the new complex therapeutic strategies. To this end, the molecular mechanisms of immune cell infiltration, functional regulation, and inflammatory cytokines during exercise must be studied more extensively and in greater depth.
